# Identifying and ranking implicit leadership strategies to promote evidence-based practice implementation in addiction health services

**DOI:** 10.1186/s13012-016-0438-y

**Published:** 2016-05-14

**Authors:** Erick G. Guerrero, Howard Padwa, Karissa Fenwick, Lesley M. Harris, Gregory A. Aarons

**Affiliations:** 1 School of Social Work, University of Southern California, 655 West 34th Street, Los Angeles, CA 90089 USA; 2 University of California, Los Angeles, Integrated Substance Abuse Programs, 11075 Santa Monica Boulevard, Suite 200, Los Angeles, CA, 90025 USA; 3 Kent School of Social Work, University of Louisville, Louisville, KY 40292 USA; 4 Department of Psychiatry, University of California, 9500 Gilman Dr. (0812), San Diego, La Jolla CA 92093-0812 USA

**Keywords:** Leadership strategies, Implementation, Organization, Management, Evidence-based practice, Managers, Addiction, Substance use disorder

## Abstract

**Background:**

Despite a solid research base supporting evidence-based practices (EBPs) for addiction treatment such as contingency management and medication-assisted treatment, these services are rarely implemented and delivered in community-based addiction treatment programs in the USA. As a result, many clients do not benefit from the most current and efficacious treatments, resulting in reduced quality of care and compromised treatment outcomes. Previous research indicates that addiction program leaders play a key role in supporting EBP adoption and use. The present study expanded on this previous work to identify strategies that addiction treatment program leaders report using to implement new practices.

**Methods:**

We relied on a staged and iterative mixed-methods approach to achieve the following four goals: (a) collect data using focus groups and semistructured interviews and conduct analyses to identify implicit managerial strategies for implementation, (b) use surveys to quantitatively rank strategy effectiveness, (c) determine how strategies fit with existing theories of organizational management and change, and (d) use a consensus group to corroborate and expand on the results of the previous three stages. Each goal corresponded to a methodological phase, which included data collection and analytic approaches to identify and evaluate leadership interventions that facilitate EBP implementation in community-based addiction treatment programs.

**Results:**

Findings show that the top-ranked strategies involved the recruitment and selection of staff members receptive to change, offering support and requesting feedback during the implementation process, and offering in vivo and hands-on training. Most strategies corresponded to emergent implementation leadership approaches that also utilize principles of transformational and transactional leadership styles. Leadership behaviors represented orientations such as being proactive to respond to implementation needs, supportive to assist staff members during the uptake of new practices, knowledgeable to properly guide the implementation process, and perseverant to address ongoing barriers that are likely to stall implementation efforts.

**Conclusions:**

These findings emphasize how leadership approaches are leveraged to facilitate the implementation and delivery of EBPs in publicly funded addiction treatment programs. Findings have implications for the content and structure of leadership interventions needed in community-based addiction treatment programs and the development of leadership interventions in these and other service settings.

## Background

According to the 2013 National Survey on Drug Use and Health, 21.6 million individuals older than 12 years old in the USA meet diagnostic criteria for a substance use disorder (SUD) [1]. In the past few decades, evidence-based practices (EBPs) have been developed and proven effective in the treatment of SUDs. Two of the most effective EBPs in SUD treatment are contingency management treatment (CMT) and medication-assisted treatment (MAT). CMT uses positive reinforcement to facilitate behavior change and has been shown to substantially improve SUD treatment adherence and reduce substance use [2–4]. A strong evidence base also supports MAT—the use of pharmacotherapies such as acamprosate for alcohol dependence, buprenorphine for opioid dependence, and naltrexone for alcohol or opioid dependence—in conjunction with psychosocial interventions [5–9]. However, despite their proven efficacy and effectiveness, neither CMT nor MAT is widely used in SUD treatment, also referred here as addiction health services [10–12].

This implementation gap is likely the result of several factors including insufficient training [13], lack of time [13], and limited program resources [13, 14]. Similarly, competing clinical priorities [15] and treatment ideologies that conflict with EBPs also inhibit implementation efforts [11, 14, 16, 17]. For the SUD treatment workforce, lack of graduate education, high provider turnover, passive leadership, and unstable funding make it difficult for publicly funded SUD programs to implement and sustain major changes to service delivery [18–20]. Given that publicly funded programs deliver the vast majority of specialty SUD treatment services in the USA [21, 22], devising and testing strategies to facilitate EBP implementation in these programs is critical to ensure that the SUD treatment population receives evidence-based care.

### Conceptual framework

Leadership behaviors are emerging as a focus of implementation science because leaders’ attitudes, priorities, and behavior [23] are increasingly being recognized as major contributors to employee and organizational outcomes [24, 25]. Leadership approaches focusing on both upper and middle managers can inform implementation strategies to help publicly funded behavioral health organizations (including SUD treatment providers) overcome barriers that inhibit EBP implementation and sustainment [26–31]. Yet most SUD program leaders are unprepared for their roles as implementers and ill equipped to effectively facilitate EBP implementation [28]. Furthermore, when SUD program leaders do successfully facilitate implementation, they are often unaware of the specific leadership approaches or strategies that they used. Rather than using formally articulated or reasoned implementation strategies, SUD program leaders use implicit leadership theories—personal cognitive constructs that guide their behaviors and interactions with staff members—to implement change [32, 33]. This is consistent with the overall management literature that highlights managers’ use of their experience, hunches, beliefs, and implicit theories to manage change [34]. Such loosely articulated approaches lack sufficient operationalization and links to theory that would allow for replication, testing, and the advancement of leadership theory applied to management and implementation science and practice.

Organizational researchers have distinguished between leadership and management behaviors while also acknowledging that there may be some overlap between the two [35, 36]. For EBP implementation, evidence suggests that leaders’ actions are essential for the creation of organizational contexts conducive to change [37, 38]. Leaders also play a critical role in facilitating implementation by carrying out management responsibilities, such as planning, organization, and supervising efforts to facilitate change [38, 39].

Most empirical research on leadership and implementation in behavioral health settings has focused on leadership style, particularly transformational leadership—a leader’s capacity to motivate staff members to change their behavior toward a particular course of action [40, 41]. Researchers have suggested that transformational leadership most likely operates through the promotion of staff development and creativity, which in turn cultivates staff openness to change [27, 42]. Another leadership style, transactional leadership, is based on exchanges in which staff members are rewarded for meeting performance targets and punished for failing to do so [40]. These two leadership styles are necessary but not sufficient to support specific employee behaviors [43].

Emerging research has also informed the development new frameworks showing that leaders need to be instrumental in guiding the implementation of new practices [44]. The implementation leadership framework developed by Aarons et al. outlines four categories of leadership behavior that support effective EBP implementation: proactive leadership (anticipating and addressing implementation challenges), knowledgeable leadership (having a deep understanding of the EBP and implementation issues), supportive leadership (supporting clinician use of EBPs), and perseverant leadership (being persistent and unwavering in EBP implementation despite challenges) [39, 45].

The present study used a staged and iterative mixed-methods approach to identify implicit leadership strategies that managers in SUD programs that employ EBPs use to implement change in their treatment organizations. We sought to determine the extent to which the identified implicit leadership strategies correspond to established leadership behaviors in the organizational and management literature. Finally, we elicited provider feedback on the perceived relative effectiveness of each leadership behavior as an implementation strategy to increase uptake of EBPs. We used CMT and MAT as examples of EBPs to provide context during these discussions. We selected these two EBPs because they represent differing treatment modalities (psychosocial vs. pharmacological), have been established as efficacious, are well recognized in the SUD treatment field, and are commonly endorsed by regulatory agencies [11]. We hypothesized that leadership strategies and concepts found in this study would be consistent with emergent work on implementation leadership in allied health care.

## Methods

### Sampling frame and recruitment

The study sampling frame consisted of 122 SUD programs that participated in a larger study of the addiction health service system in Los Angeles County, CA, USA, that is described in greater detail elsewhere [12]. To ensure that study participants worked at programs that were viable and had successfully implemented an EBP, the study sample was narrowed to 60 programs that had been in operation for at least 5 years and had demonstrated the use of EBPs such as CMT and MAT. Verification of EBP delivery relied on survey responses and reports during site visits that were part of the larger study. Because program size is associated with EBP implementation in SUD treatment programs [12, 46, 47], a mix of 12 large and small programs was purposively selected from the group of 60 programs that met study inclusion criteria. From each of these 12 programs, one program director and one clinical supervisor were contacted and invited to participate in the study. In several smaller programs, the same individual filled both program director and clinical supervisory roles, so only one individual was recruited from those programs. The final sample included 18 individuals—7 who described themselves as clinical supervisors and 11 who described themselves as program directors. Half of the managers had a master’s degree, and their average experience in the field was 15 years. Managers supervised an average of five staff members (see Table 1).

**Table 1:** Characteristics of managers (*N* = 18)

Variable	*M* (SD) or %
Age, years (range, 50–73)	46 (8.5)
Female	72
Race and ethnicity	
White	33
African American	33
Hispanic	33
Education	
Some college	28
Bachelor’s degree	5
Master’s degree	50
Doctoral degree	17
Experience in substance abuse treatment, years	15.3 (9.9)
Number of staff members supervised	5.1 (3.6)
Number of clients served per month	147.5 (137.9)
Program offers contingency management treatment (often or always)	44
Program offers medication-assisted treatment (often or always)	33

This study was approved by the University of Southern California Institutional Review Board (No. UP-14-00105), and all recruited individuals agreed to participate in our study and provided informed consent. Each participant received a $50 gift card at the conclusion of each focus group or interview. Focus groups and interviews were audio recorded, videotaped, and professionally transcribed for analysis.

### Procedures

**Fig. 1:**
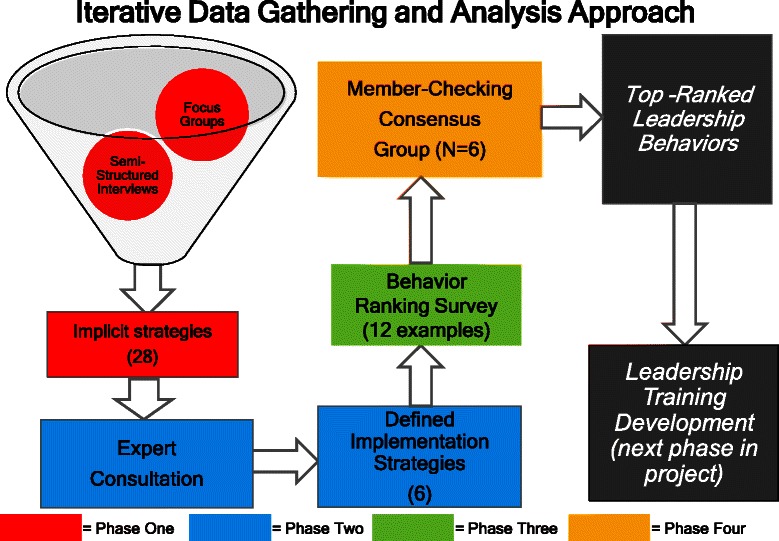
Iterative data gathering and analysis approach

The study relied on a hybrid modified Delphi approach, using a mixed-methods iterative process to gather and analyze data. Specifically, focus groups, semistructured interviews, expert consultation, surveys, and multiple analytic methods were used to gather data and inform subsequent steps (see Fig. 1). Participants went through the following four phases to identify, evaluate, and rank leadership behaviors that could be considered as strategies for implementation of evidence-based addiction treatment practices.

### Phase 1: focus groups, semistructured interviews, and analysis

Two weeks before the study began, participants received a questionnaire designed to orient them to the study, encourage them to reflect on their leadership behaviors, and prepare them to discuss issues related to leadership and the implementation of EBPs [48]. Each participant then took part in one of four 2-h focus groups (two for clinical supervisors, two for program directors) facilitated by three PhD-level researchers (EG, HP, and LH) with three or more years of training and experience conducting qualitative research. Focus groups elicited participant experiences with EBP implementation, strategies used to implement new practices, and practical approaches used to facilitate change in their organizations. Participants were prompted to describe strategies specific to each implemented EBP. Discussion was not restricted only to CMT and MAT, and managers were encouraged to talk freely about implementation strategies more generally.

Two to four weeks after the focus groups were completed, each participant engaged in an individual semistructured phone interview with one of two qualitative researchers (LH and HP). During the interviews, researchers asked participants to expand on the information they shared during the focus groups and provide more in-depth information about their individual implementation strategies, opinions about EBPs, and approaches to facilitating change. Interviews ranged from 30 to 70 min in length and were recorded and professionally transcribed for analysis.

Focus group and semistructured interview transcripts were deidentified and uploaded to ATLAS.ti (7.0) to facilitate data management and analysis. The two qualitative researchers read each focus group and interview transcript independently. Using constructivist grounded theory techniques, the researchers conducted open coding, focused coding, and axial coding to identify major themes and categories and determine relationships between them [49]. The two researchers met regularly throughout the analytic process to discuss similarities and differences in coding, reconcile differences in interpretation, and ultimately reach 100 % consensus. The coding resulted in a list of 28 implementation and leadership behaviors. The two researchers analyzed transcripts to determine if any of the 28 behaviors were more or less common among program directors or clinical supervisors or among individuals from large or small programs. No discernible differences were found in the content or frequency of strategies mentioned by individuals based on job role or organization size.

### Phase 2: expert consultation and implementation strategy definition

After the list of 28 leadership behaviors was compiled, the research team shared the list with an expert implementation science consultant (GA) [30, 50], who organized the 28 leadership behaviors into six broad leadership strategies corresponding to leadership behaviors from organizational and management research literature. For each of the six broad leadership strategies, the expert consultant then chose two behaviors from the original list of 28 leadership behaviors that he determined to best exemplify each leadership strategy. The product of this process was a list of six broad leadership strategies, with 12 specific leadership behaviors that were exemplars of each of the six strategies.

### Phase 3: ranking survey

All study participants were invited to rate the 12 specific behaviors that were identified from the second phase in an online survey. Participants were asked to rate behaviors on a Likert scale based on their perceived utility (“This is a useful strategy”), feasibility (“I believe I will be able to implement this strategy”), relevance to their program (“This strategy is relevant to my program”), cost-effectiveness (“This strategy would be cost effective for my program”), and expected impact on staff behavior (“This strategy will have an impact on my staff”). The rating scale was as follows: 1 = strongly disagree, 2 = disagree, 3 = neither agree nor disagree, 4 = agree, and 5 = strongly agree. Participants had approximately 30 days to respond and received a $50 gift card upon completion of the ranking survey. Survey data were analyzed using SPSS analytic software to generate frequencies including means, medians, and standard deviations.

For each strategy, mean ratings for each of the five categories (usefulness, feasibility, relevance, cost-effectiveness, expected impact) and an overall rating for each strategy were derived. To offset issues inherent in using means as measures of central tendency, medians and standard deviations for each item were also analyzed. The research team also analyzed data to determine if there were differences in response patterns between program directors and clinical supervisors or between respondents from large and small programs. In both cases, differences were minimal (results available upon request).

### Phase 4: member-checking focus group

To further explore and verify findings from the survey, we conducted a member-checking focus group [51, 52] with six study participants. These participants were purposively selected for participation to have maximal variation based on program size and survey responses. Three researchers (EG, HP, and LH) facilitated the focus group by presenting findings from the preceding study steps to participants, eliciting participant feedback on study findings, and facilitating discussion to resolve issues that remained unclear and discrepancies in the data. Consensus group protocols are available upon request from the corresponding author.

The member-checking focus group was recorded and transcribed and transcripts were uploaded to ATLAS.ti (7.0) for data management analysis. Two researchers (HP and LH) read the focus group transcript independently and used constructivist grounded theory techniques to conduct open coding followed by focused coding and axial coding to identify major themes and categories and determine relationships between them [49]. The two researchers met regularly throughout the analytic process to discuss similarities and differences in coding, reconcile differences in interpretation, and reach 100 % consensus on the interpretation of the member-checking focus group transcript. Interpretation of findings was adjusted in light of information from analysis of the member-checking consensus group.

## Results

### Phase 1 results

As shown in Table 2, analysis of the transcripts from the focus groups and the semistructured interviews that followed yielded a list of 28 distinctive leadership behaviors. Participants reported using these leadership behaviors to facilitate the implementation of new practices in their treatment organizations. However, managers were not able to connect their leadership behaviors to implementation with a body of literature, framework, or personal theory. As one clinical supervisor explained when asked what strategies she uses to facilitate implementation, “My strategies have no names. … I just do what works.”

**Table 2:** Implicit leadership behaviors organized into six broad categories exerted from focus groups and semistructured interviews (phases 1 and 2)

Category	Leadership behaviors
Demonstrating knowledge	1. Formally demonstrate a new intervention with a client in front of staff.
	2. “Jump in” to a session and take over for staff to show them how to implement a new intervention.
	3. At a staff meeting, bring in a client who benefited from receiving a new intervention to talk about their experience, explain how it helped them in their recovery.
	4. Record sessions or groups where staff deliver a new intervention, then review recording during supervision or group discussion in order to coach staff.
Proactively facilitating implementation	5. Formally train staff about a new intervention and why it works.
	6. Ask staff about challenges they face when working with clients (e.g., poor attendance at groups, difficulty managing cravings) and then teach staff how new interventions will help address these challenges.
	7. Give staff tools to track client programs during the course of a new intervention to prove that it works (e.g., for an intervention to increase group attendance, give staff a chart to track how often clients show up).
	8. Tell staff organizational leadership is invested in implementing a new practice
Proactively creating a climate conducive to implementation	9. Hire staff who are receptive to change and a good fit for the organization.
	10. Fire staff who do not implement change or threaten discipline if changes are not implemented.
	11. Designate a staff member who is well-suited to implement a new practice as a “champion” for change.
	12. Inform staff that changes need to be made since they are being mandated by outside funders.
Supporting change through individualized connections	13. Talk to staff about how you were “once in their shoes” and how you have done the work you are asking them to do; empathetically explain you know how challenging it can be.
	14. Have an “open door” policy and always be available for staff if they have questions or concerns about a new intervention.
	15. Ask staff what help or support they need to deliver a new intervention.
	16. Assist staff with other duties (e.g., paperwork) while they adjust to delivering a new intervention.
	17. Encourage staff self-care (e.g., tell them to take a vacation day) so they feel refreshed when implementing a new intervention.
Supporting change through transactions	18. Give staff small gifts (trinkets, stationary) as reward for implementing a new practice.
	19. Give staff large gifts (jewelry, a trip) as reward for implementing a new practice.
	20. Give staff promotions or salary increases as reward for implementing a new practice.
	21. Praise staff for implementing a new practice.
	22. Chastise or discipline staff who do not implement a new practice.
Perseverance through problem-solving	23. Talk to staff to identify reasons for resistance and reframe implementation of new practices (e.g., tell them a new practice is not a “change in how we do things” but “adding something new to the services we offer.”).
	24. After teaching staff about a new intervention, elicit feedback on how intervention can be improved, use this information to adjust intervention.
	25. Encourage staff to adapt new interventions to fit within the work they are already doing with clients.
	26. Use alternative funding sources (grants, donations) to implement new practices.
	27. Use flexible funding sources to support the implementation of new practices.
	28. Collaborate with outside agencies and have them deliver new practices instead of in-house staff.

### Phase 2 results

A distillation of the 28 strategies that emerged from phase 1 resulted in six broad implementation leadership strategies. See Table 2 for behavioral examples of these strategies.

#### Demonstrating knowledge

This strategy involved program leaders having a deep understanding of new practices and potential issues that could affect EBP implementation. The two management behaviors mentioned in focus groups and semistructured interviews that were most representative of this strategy were role modeling (i.e., performing an intervention in front of staff as a demonstration and jumping into a session to model an innovation for staff). As one clinical supervisor explained, by “doing what I expect them [staff members] to do with the patients, they see what I’m telling them [to do]” and they learn that it is effective because they have “seen it.” Moreover, role modeling allows staff members to observe clinical skills in action, by giving them an opportunity to “sit and see how it goes from a different perspective.” This dimension is akin to knowledgeable leadership as described by Aarons et al. [39] and one dimension of transformational leadership known as inspirational motivation [40, 53] which describes a leader’s ability to engender buy-in and enthusiasm for a course of action.

### Proactively facilitating implementation

This strategy involved leaders developing plans to facilitate implementation, removing obstacles to implementation, and establishing clear standards for a staff to follow when implementing new practices. The two management behaviors mentioned in focus groups and semistructured interviews that were most representative of this strategy involved removing staff-level barriers to implementation by formally training staff members about new practices and educating them on how new practices can address challenges they face. To convince staff members to make changes, one clinical supervisor explained, it is critical to educate them about “why it works, why it is that I wanted to do this rather than what they’ve been doing so far.” Moreover, by educating staff members on how new practices can address challenges they face, one director elaborated, leaders can minimize resistance to change by showing “how where we want to go is going to be a benefit to them” and help “get the buy in” needed to implement and sustain change. As a form of proactive leadership, these behaviors are consistent with being able to articulate elements of EBPs and how they lead to patient behavior change, as described by Aarons et al. [39].

#### Proactively creating a climate conducive to implementation

This dimension involves leaders using their authority to make staffing decisions to facilitate implementation. The two behaviors mentioned in focus groups and semistructured interviews that were most representative of this strategy were hiring staff members who are receptive to and able to handle change and informing staff members that if they do not implement change they could lose their job. Staff flexibility and adaptability are critical, one director elaborated, because as knowledge about addiction advances and new interventions are developed, it is essential to “have a group of people who are willing to grow” and make significant changes to the services they deliver. Conversely, staff members who are unwilling or unable to adapt are likely to hinder the implementation of new practices. By framing the implementation and sustainment of new practices as a performance issue, one director explained, leaders can ensure that the importance of adaptability is clear to the staff. This approach is akin to proactive leadership described by Aarons et al. [39] and a dimension of implementation climate (selection for openness) described by Ehrhart et al. [54].

#### Supporting change through individualized connections

This strategy involves recognizing and appreciating staff implementation efforts and taking steps to support staff efforts to learn about and use EBPs. The two behaviors mentioned in focus groups and semistructured interviews that were most representative of this strategy were having an open-door policy with the staff and asking staff members what could support them in implementing EBPs. Having an open-door policy is critical because it provides opportunities for supervision and guidance on how to implement new practices when needed. “These require practice, like doing a pushup or a cartwheel,” one supervisor explained. “You have to have somebody who’s coaching you to do it correctly.” Similarly, asking staff members what can support them in implementing new practices can facilitate change because it ensures that staff members receive the assistance they need and feel that they “have a say … a voice” in how new practices are implemented. This dimension represents a form of supportive leadership described by Aarons et al. [39].

#### Supporting change through transactions

This strategy involves leaders taking actions to incentivize change through the use of positive reinforcements. The two practices mentioned in focus groups and semistructured interviews that were most representative of this strategy were giving small token gifts to reward the implementation of a new practice or giving promotions or rewards for implementation. “Things like this,” explained one supervisor, “show the value of the person [staff member].” By helping improve morale, they also create work climates that are more conducive to change. These practices clearly represent the key concept of transactional leadership, whereby leaders rely on rewards to promote performance [41, 53], and also reflect supportive leadership approaches [39].

#### Perseverance through problem solving

This strategy involves leaders strategically reacting to challenges that arise during implementation and persevering through successes and setbacks. The two behaviors mentioned in focus groups and semistructured interviews that were most representative of this strategy were using alternative funding sources and flexible funding approaches to support the implementation of new practices. This dimension is consistent with perseverant leadership as described by Aarons et al. [39].

### Phase 3 results

Table 3 presents the means and standard deviations for the overall ratings of each of the 12 leadership behaviors listed in the rating survey. The three highest-rated behaviors were (a) hire staff members who are receptive to and able to handle change (M = 4.71; SD = 0.52), (b) have an open-door policy with the staff (M = 4.46; SD = 0.63), and (c) ask staff members what supports their ability to deliver interventions (M = 4.41; SD = 0.60). The three lowest-rated strategies were (d) jump into a session and take over for the staff (M = 3.56; SD = 1.24), (e) inform staff members that if they do not implement change they may lose their job (M = 3.68; SD = 0.97), and (f) give staff members promotions or salary increases as a reward for implementing a new practice (M = 3.83; SD = 0.90). Due to the small sample size, we were unable to conduct statistical significance testing. However, we observed the following patterns in our descriptive statistics: As evidenced by examination of the standard deviations, there was less variability among leaders regarding the top-ranked strategies compared to the lower-ranked strategies; the standard deviation for the top-ranked strategy (0.52) was less than half that of the lowest-ranked strategy (1.24). There was little difference between overall mean ratings and mean ratings by category (usefulness, feasibility, relevance, cost-effectiveness, and expected impact). There was also little difference between ratings given by program directors and those given by clinical supervisors.

**Table 3:** Results of leadership ranking survey (*n* = 12) (phases 3 and 4)

Rank	Behavior^a^	Strategy^b^	Type^c^	M	SD
1	Hire staff members who are receptive to and able to handle change	Proactively creating a climate conducive to implementation	Proactive	4.71	0.52
2	Have an open-door policy with staff	Supporting change through individualized connections	Supportive	4.46	0.63
3	Ask staff members what supports their ability to deliver interventions	Supporting change through individualized connections	Supportive	4.41	0.60
4	Perform an intervention in front of staff as a demonstration	Demonstrating knowledge	Knowledgeable	4.40	0.63
5	Teach staff how the intervention helps clinicians address challenges they face	Proactively facilitate implementation	Proactive	4.25	0.71
6	Formally train staff about an intervention and why it works	Proactively facilitate implementation	Proactive	4.25	0.60
7	Manage change creatively by using community or charitable resources	Perseverance through problem solving	Perseverant	4.09	0.73
8	Manage change by using flexible funding	Perseverance through problem solving	Perseverant	3.96	0.74
9	Give small token gifts to reward implementation	Supporting change through transactions	Supportive	3.94	1.11
10	Give promotions or rewards for implementation	Supporting change through transactions	Supportive	3.83	0.90
11	Inform staff members that if they do not implement change they may lose their job	Proactively creating a climate conducive to implementation	Proactive	3.68	0.97
12	Jump into a session and take over for staff	Demonstrating knowledge	Knowledgeable	3.56	1.24

^a^Behavior refers to manager’s behavior related to employees

^b^Strategy refers to manager’s selected approach to engage employee in implementation efforts

^c^Type refers to Implementation Leadership Scale categories (Aarons et al. [39])

Three of the top six leadership behaviors were forms of proactive leadership and two of the top three leadership behaviors were forms of supportive leadership, as identified by Aarons et al. [39]. Behaviors indicative of proactive leadership were ranked both relatively high (hire staff members who are receptive to and able to handle change, ranked no. 1) and low (inform staff members that if they do not implement change they will lose their job, ranked no. 11), as were behaviors indicative of knowledgeable leadership (perform an intervention in front of staff members as a demonstration, ranked no. 4; jump into a session and take over for the staff, ranked no. 12). The proactive and supportive practices had some congruence with aspects of transformational leadership [40, 53].

### Phase 4 results

In the member-checking consensus group, participants elaborated on why they considered the two highest-ranked strategies (having an open-door policy; hiring staff members who are open to change) to be the most effective. They also described why they considered the two lowest-ranked strategies (punishing, threatening, or terminating employment; role modeling by jumping into a session) to be the least effective ways to facilitate change.

#### Having an open-door policy with the staff

Consensus group members explained why they believed that having an open-door policy with staff was a particularly effective way to facilitate the implementation of new practices. Being constantly available, consensus group members elaborated, serves two interrelated functions. First, it provides the staff with a sense of support and reinforces the message that the entire organization is invested in seeing staff members succeed in implementing a new practice. “[If you say,] ‘The door is open, come in,’ you encourage it,” explained one director, “so that staff [members] continue to feel comfortable coming in and asking those questions and gaining positive reinforcement,” which encourages them to continue implementing changes. Moreover, consensus group participants reported that by having an open-door policy, they are able to provide staff members with what one director called “on-the-job training … because lots of people are able to come to you the instant they need you.” Consistent availability, he concluded, is a good way to help ensure that staff members implement new practices correctly and effectively.

#### Hiring staff members who are receptive to and able to handle change

Consensus group participants reported that staffing is the most important factor in facilitating or hindering the implementation of new practices in their treatment organizations, particularly because of ongoing changes in the SUD treatment field. As one clinical supervisor explained:

The science of addiction medicine and treatment is really changing, continuously … so you have to have a mind that is willing to learn and improve and change with the science, change with the industry. If you’re not receptive to that, then you’re really going to interfere … with the organizational needs. … [So] when you’re hiring, you have to consider … How well do they adapt to changes? How much are they willing to learn?”

In addition to emphasizing the importance of hiring staff members who are receptive to change, consensus group members reported that the shifting nature of the addiction field has made them more aware of the importance of hiring staff members whose background and training fits with the newest professional standards. Traditionally, explained one director, programs predominantly hired “folks who had completed the program” as clients recovering from drug or alcohol addiction. However, these former clients are now expected to go through additional training prior to being hired to make them a better fit for the current SUD treatment environment, which has become increasingly oriented toward evidence-based care.

[In the past,] we were saying, “We’ve got to give these folks an opportunity [to work by hiring them], let’s put them in the field [right away].” … But now we’re saying, “OK, you need a little bit more training [after completing treatment]. You need to go to school a bit longer.” … We’re taking a step back and saying, “We know you’ve completed treatment and we know we want to give you an opportunity, but wait, let’s give you a little bit more … education.”

Consequently, as the field of SUD treatment becomes increasingly scientifically oriented, consensus group participants reported that their organizations are prioritizing hiring staff members with more formal training in different EBPs and flexibility to adapt to an ever-changing healthcare environment.

#### Punishing, threatening, or terminating employment

Participants elaborated why they ranked the use of punishment or threats low on the ranking survey. “When you give someone an ultimatum,” explained one director, “that shuts people down” and makes them focus on just implementing a practice rather than implementing it correctly or well. “They may … do what you’re asking them to, but they’re going to be sour about it and they’re going to [have] an ugly way of doing it.” Furthermore, this director elaborated, if a staff member implements a practice poorly, that individual could become what he termed “the bad apple in the bunch” and “infect other staff [members]” with negative attitudes about both the new practice and the organizational leaders who are mandating implementation. Another director agreed, pointing out that much like threatening clients is an ineffective way to get them to stop using drugs or alcohol, threatening staff members if they do not make desired changes can be counterproductive:

Punishment doesn’t work. … It sends a negative statement from the beginning. If you want to get people to make a change, you provide them with the necessary skills they need to implement the change. You don’t tell them that, “If you don’t do it, this is an ultimatum.” That doesn’t work in any field, to give people ultimatums, because naturally, people get defensive and then they want to go against the grain, [saying] “Who are you to give me an ultimatum to do X, Y, and Z?”

#### Role modeling by jumping into a session

Consensus group participants explained that they believed jumping into a session to demonstrate an intervention was a poor strategy because it could implicitly undermine staff’s authority when working with clients. Although some study participants endorsed this approach, consensus group members explained that it would be difficult to spontaneously take over a session without, as one director elaborated, “diminishing that staff member” and communicating that “what they’ve learned or what they’re bringing to the table” is somehow insufficient. Although they believed that demonstrating interventions in front of the staff could be beneficial, consensus group participants explained that the potential drawbacks of doing this in a spontaneous or disruptive manner could potentially outweigh the benefits.

## Discussion

This study identified implicit leadership strategies that managers in SUD treatment programs in Los Angeles County, one of the largest treatment systems in the USA, use to implement EBPs in their organizations. This study also linked these implicit strategies to two leadership styles (transformational and transactional leadership) [27, 40, 42] and one type of leadership approach to EBP implementation—knowledgeable, proactive, supportive, and perseverant [39, 45]. Because identifying strategies to facilitate EBP implementation in publicly funded programs is critical to ensure that the SUD treatment population receives evidence-based care, findings from this study will help improve practice while also helping refine loosely articulated management approaches to implementation, operationalizing and linking organizational concepts to theory to improve replication and testing, and advancing leadership and implementation science theory.

We determined how the leadership behaviors used by SUD program managers correspond to established leadership strategies from the organizational and management literature. We accomplished this by relying on a rigorous methodology to elicit provider feedback on the perceived relative effectiveness of different leaders’ behaviors that could lead to effective implementation approaches. Using a multifaceted approach to identify, rank, and evaluate strategies, we found that the most effective strategies for implementation of EBPs align with an emerging model of leadership implementation [30]. This model highlights leadership behaviors that are proactive to respond to implementation needs, supportive to assist the staff during the uptake of a new practice, knowledgeable to properly guide the implementation process, and perseverant to tackle ongoing barriers that are likely to stall the implementation effort.

These strategies focus on leaders’ behaviors in relation to followers’ engagement in implementation efforts as the main locus of influence. Strategies identified as highly effective focus on fostering nurturing relationships between program leaders and staff while also targeting specific goals (EBP implementation). This is similar to the strengths-based approach commonly used in health care, which focuses on developing the resources and abilities of clients through a supportive provider-client relationship to promote positive health behavior change [55, 56]. This feature of leadership among managers of SUD treatment is logical considering that most managers in this field emerge from clinical practice, where they rely on the provider-client relationship to promote client growth [57, 58]. However, the degree to which this phenomenon is more common in SUD treatment services than other human services remains unclear.

This strengths-based approach may also explain why managers’ ratings of the strategies differed based on the framing of behaviors. If the overall approach undermined staff members (e.g., firing someone for not engaging in desired behavior), managers ranked it as ineffective, whereas if a strategy was positively framed (e.g., rewarding desired behavior), managers considered it highly effective. This finding has implications for the application of two of the most researched aspects of leadership—transformational and transactional leadership—as they related to the implementation of new practices in addiction treatment settings. Our results align with recent research indicating that transformational leadership, in which leaders develop staff talents and promote staff growth, is an efficacious approach during implementation [27, 37]. In addition, dimensions of transactional leadership based on positive reinforcement, such as contingent rewards [40], can be effectively applied to EBP implementation. However, some of the dimensions of transactional leadership, such as active management by exception, in which strong corrective action is given when staff behavior deviates from the target behavior, may not fit with the values of SUD treatment managers. Thus, a transactional leadership approach should be applied in a careful and measured way for this treatment setting [59].

The leadership strategies identified here are not foreign to healthcare services [33, 59], but they have not yet been researched in the context of addiction health services and the delivery of evidence-based SUD treatments. Managers’ recognition of these strategies to implement new EBPs is a contribution of this study. These strategies (e.g., strategic hiring, asking about staff needs, and establishing an open-door policy) will be critical to implementation efforts in the addiction health service field, which is currently professionalizing and developing its workforce.

The extent to which these strategies may be more effective for implementation if used in concert or individually remains an empirical question that needs exploration. Emerging research has suggested that using strategies such as establishing an open-door policy is a passive but important step to enhance communication [60]. Employees are much more likely to be forthcoming when their input is solicited and they feel safe [60]. Hence, also using the strategy of asking staff members what they need to effectively implement a new practice may be a more proactive approach to ensuring action. Environments where staff are comfortable asking questions, challenging current practices, and making mistakes are critical for implementation because they may enhance employee commitment to change and willingness to implement new healthcare practices [25]. Overall, approaches managers can use to create environments conducive to EBP implementation are teachable and their uptake among SUD treatment managers is certainly feasible.

### Implications

Our results have implications for developing leadership interventions in SUD treatment programs. Managers have significant influence on their staff, and staff members have a great need for support and professional development in the addition field [23–25]. The small size of many community-based treatment programs (two to three counselors), limited professional development [12], daily and direct interaction with managers, and shared service goals may allow leadership interventions to have a significant impact on a leader’s behaviors and the staff’s subsequent implementation behaviors. However, prior research has indicated that the leadership capacities of directors and supervisors in addiction treatment settings is highly variable [28], so resources are needed to enhance the capacity of program managers to invest in their leadership development. Leadership development interventions can be informed by the explicit leadership strategies identified in this study.

The present findings also have implications for improving implementation efforts and client outcomes in addiction treatment programs. Leadership strategies can enhance the capacity of addiction health services programs to adopt EBPs, particularly to improve staff attitudes toward, use of, and fidelity to EBPs. These strategies can also be tailored to each organization’s context to develop organizational readiness for implementation of change. Finally, the iterative multimethod approach used here to identify and rank implicit leadership strategies for implementation may have implications for future research. Our methodological approach can help researchers identify context-dependent leadership behaviors that may enhance implementation efforts.

### Limitations

Some limitations of the present study should be noted. The study used a relatively small sample of providers to obtain a deep understanding of implicit strategies to implement EBPs in urban outpatient SUD treatment programs. Although derived from a small sample, the qualitative data were not intended to be representative of the entire addiction health services system in the USA. However, our purposive sampling strategy to use small and large programs improved generalizability, and the themes that emerged from this work are representative of key features highlighted in the literature [61–63]. Furthermore, the comprehensive and multimethod approach to data collection used in this study is consistent with other research in behavioral health [30, 64, 65]. Our results did not provide information about implementation strategies tailored to specific EBPs, but rather to implementation of EBPs generally. Future studies can build on our results and address these limitations by examining these strategies as they relate to other locations, broader service contexts, and specific EBPs.

## Conclusions

The results of this study suggest that certain leadership strategies are implicitly used to facilitate EBP implementation in SUD treatment programs. Results from this study match other accepted leadership approaches. Managers’ strategies aligned with proactive, supportive, knowledgeable, and perseverant approaches, dimensions that fit with transformational and transactional leadership styles. There is increasing evidence that managers need to develop leadership behaviors that promote specific employee change behavior [44]. This suggests that targeted professional development and task-oriented orientations may be promising approaches to develop leadership capacity for implementation. In particular, one way to train leaders is to make implementation leadership strategies explicit and then link leader experiences back to theory [66]. Study findings should empower managers to move from “just doing what works” to identifying, measuring, and managing the extent to which their strategies influence EBP implementation. Overall, findings contribute to the emerging literature on identifying new and refining existing strategies to build practitioners’ capacity to implement EBPs in community-based healthcare settings [67, 68].

### Ethics approval and consent to participate

This research was performed in accordance with the Declaration of Helsinki and approved by the Institutional Review Board at University of Southern California (no. UP-14-00105). Informed consent to participate in the study was obtained from participants.

## Abbreviations

CMT: contingency management treatment
EBPs: evidence-based practices
MAT: medication-assisted treatment
SUD: substance use disorder
